# Approach to minimally invasive donor hepatectomy: Laparoscopic, robotic, or bit of both!

**DOI:** 10.1002/ags3.12672

**Published:** 2023-03-23

**Authors:** Christi Titus Varghese, Biju Chandran, S. Sudhindran

**Affiliations:** ^1^ Department of Gastrointestinal Surgery and Solid Organ Transplantation Amrita Institute of Medical Sciences and Research Centre Kochi India

**Keywords:** donor hepatectomy, laparoscopic, minimally invasive, robotic

The advent of living donor surgery has been an important milestone in the evolution of liver transplantation over the past two decades. This accomplishment has been built on the twin pillars of utmost donor safety and exemplary recipient outcomes. Currently, mortality to the donor is <0.5%. However, morbidity that is predominantly wound related, which occurs in approximately 30%–38% of cases, remains a pressing concern. It is against this backdrop that minimally invasive donor hepatectomy (MIDH) garnered interest. There is still debate as to what constitutes MIDH. In this article we restrict the term MIDH to totally laparoscopic or robotic resection and use of a remote incision, usually Pfannenstiel for graft retrieval.

The first laparoscopic live donor hepatectomy (LLDH) for left lateral segmentectomy was performed in 2002. It was not until a decade later that laparoscopic right donor hepatectomy as well as the first robotic live donor hepatectomy (RLDH) were completed. To date about 1000 LLDHs and a similar number of RLDHs have been reported. Donor and recipient outcomes following MIDH were comparable with open donor hepatectomy, notably with no increase in postoperative complications. Additionally, lesser blood loss, reduced pain scores, and earlier hospital discharge leading to faster return to work were observed as plausible benefits of MIDH. This has led to multiple consensus conferences on MIDH and they have recommended minimally invasive left lateral segmentectomy as an acceptable alternative to its open counterpart.^1^

Liver mobilization without trauma, control of bleeding during transection, and handling of hepatic duct are a few of the hurdles that need to be overcome while advancing from open to MIDH. The majority of centres well‐versed in living donor liver transplantation (LDLT) usually have negligible experience in minimally invasive surgery and vice versa. Furthermore, units with experience in LDLT as opposed to those with expertise in minimally invasive surgery were able to successfully establish MIDH programmes, affirms the importance of having a successful LDLT programme as a basic tenet to performing MIDH. In this context, the evident question is whether the robotic platform or laparoscopy will be the superior option. Although laparoscopy with its lower establishment costs, widespread availability, and conventional accessories (cavitron ultrasonic surgical aspirator [CUSA]/water jet, fluorescent radiography) appears to be the natural choice, a closer scrutiny of published data suggests otherwise. Despite its early start, LLDH has had a slower assimilation among transplant surgeons when compared to RLDH. Reasons are manifold. Firstly, versatility of the robotic arm with seven degrees of motion “mimicking open surgery,” tremor filtering with motion scaling, ability to perform precise suturing, abolition of fulcrum effect, and superior ergonomics has resulted in a shorter learning curve for RLDH when compared to LLDH.^2^ Secondly, most of the reports of MIDH have revealed outcomes in carefully selected donors with uncomplicated anatomy while data on donors with complex anatomy (large grafts, inferior hepatic veins requiring reconstruction/portal/biliary, and arterial variations) is lacking. Such extended‐criteria donors do constitute a substantial portion of live donors, where robotic platform, with its capability to control bleeding in deeper planes and tackle biliary variations with accuracy, may possibly offer several advantages over laparoscopy. Indeed, centres with the robotic platform have expanded their selection criteria early on to include all donors suitable for open donor hepatectomy.^3^ Thirdly, laparoscopy is labour intensive with the need to train multiple members to initiate and successfully perform MIDH. Availability of a dual console for training and the reduced dependence on other team members except for a bedside surgeon makes proctoring easier on the robotic platform. Conversely, drawbacks of the robot cannot be ignored and include lack of haptic feedback, non‐availability of CUSA/water jet, limited number of ports, time‐consuming instrument exchange, fixed table position, and significantly higher costs when compared to open/laparoscopic donor hepatectomy.

Multiple studies have already enumerated that RLDH has several advantages when compared to open surgery. Interestingly, a new study on robotic surgery for hepatocellular carcinoma has reported reduced incidence of post hepatectomy liver failure, less hospital stay, and oncological outcomes comparable to open resection as well.^4^ However it must be noted that, head‐to‐head comparisons between robotic and laparoscopic surgery is lacking. A recent comparison between LLDH and RLDH revealed that while RLDH takes longer, complex anatomy was more common in the RLDH group.

How can a unit performing open donor hepatectomy move towards MIDH? Requirement of a proctorship period under an experienced mentor performing MIDH is non‐negotiable. Additionally, selection of donors suitable for MIDH and low threshold for conversion at the start of the programme will help the team in adapting to the technique without jeopardizing patient outcomes. The surgical robot at many centers is utilized by multiple specialities. Restricting the use of the robot exclusively for MIDH or procuring multiple robotic platforms may not be financially viable. This is likely to lead to longer waiting lists and prolonging the time needed by a novice team to negotiate the learning curve. Although multiple industry vendors are in the process of introducing cheaper surgical robots, a delay before it is approved and freely available is expected. Conversely, the laparoscopic system at most of the institutions remains underutilized for MIDH due to the technical complexity involved. Lately, a study has suggested that the learning curve for LLDH is minimal after mastering RLDH.^5^ Whether performing LLDH on uncomplicated donors and selectively using the robotic platform to do RLDH on donors meeting the extending criteria needs further scientific rigor. This could lead to better rationalization of resources without compromise on patient outcomes.

As use of technology and awareness grows among surgeons, it is not unreasonable to believe that MIDH will become the mainstay in the future. However, randomized studies on short‐term as well as long‐term outcomes are lacking. As randomized trials may logistically and perhaps ethically be impossible, it is imperative that all centers performing MIDH share data so that guidelines are further streamlined. As we continue to innovate, it is worth remembering that any exposure of the donor to surgical risks is in violation of the basic tenet of medicine, “primum non nocere.”

## FUNDING INFORMATION

None.

## CONFLICT OF INTEREST STATEMENT

The authors declare no conflicts of interest for this article
